# Effect of Intraluminal Thrombus Burden on the Risk of Abdominal Aortic Aneurysm Rupture

**DOI:** 10.3390/jcdd10060233

**Published:** 2023-05-26

**Authors:** Aykut Can Arslan, Huseyin Enes Salman

**Affiliations:** Department of Mechanical Engineering, TOBB University of Economics and Technology, Ankara 06530, Turkey; aykutcan.arslan@etu.edu.tr

**Keywords:** computational fluid dynamics, abdominal aortic aneurysm, fluid–structure interaction, intraluminal thrombus, rupture, mechanical stress, ADINA

## Abstract

Abdominal aortic aneurysm (AAA) is a critical health disorder, where the abdominal aorta dilates more than 50% of its normal diameter. Enlargement in abdominal aorta alters the hemodynamics and flow-induced forces on the AAA wall. Depending on the flow conditions, the hemodynamic forces on the wall may result in excessive mechanical stresses that lead to AAA rupture. The risk of rupture can be predicted using advanced computational techniques such as computational fluid dynamics (CFD) and fluid–structure interaction (FSI). For a reliable rupture risk assessment, formation of intraluminal thrombus (ILT) and uncertainty in arterial material properties should be taken into account, mainly due to the patient-specific differences and unknowns in AAAs. In this study, AAA models are computationally investigated by performing CFD simulations combined with FSI analysis. Various levels of ILT burdens are artificially generated in a realistic AAA geometry, and the peak effective stresses are evaluated to elucidate the effect of material models and ILT formation. The results indicate that increasing the ILT burden leads to lowered effective stresses on the AAA wall. The material properties of the artery and ILT are also effective on the stresses; however, these effects are limited compared to the effect of ILT volume in the AAA sac.

## 1. Introduction

Abdominal aortic aneurysm (AAA) is the dilatation of abdominal aorta greater than 50% of its normal diameter [1,2]. The enlargement of the aortic segment results in severe alterations in flow hemodynamics and arterial mechanics [3,4,5]. Unless treated, the aneurysm continues to grow, and catastrophic mechanical failure which is known as AAA rupture may take place. AAA rupture is a health emergency in which 80% of ruptured AAAs result in death [6].

From a mechanical point of view, the rupture occurs when the flow-induced hemodynamic forces exceed the limit that the arterial wall can withstand [7,8]. The flow-induced forces on the arterial wall cannot be directly determined by advanced imaging modalities such as magnetic resonance imaging (MRI) or computed tomography (CT). At that point, computational methods such as computational fluid dynamics (CFD) simulations and fluid–structure interaction (FSI) analysis provide an opportunity to model the flow and determine the flow-induced hemodynamic forces in a virtual environment by using patient-specific geometries [9,10].

The computational modeling of AAA is a detailed process that requires patient-specific geometry to be extracted by medical imaging tools, followed by solving the governing physical equations using appropriate methodology. Therefore, the entire modeling process requires a certain amount of time on the scale of days.

The findings of CFD studies report that the flow velocities are relatively slower in the enlarged AAA sections [11,12,13]. As a result of the nearly stagnant flow in the aneurysm sac, wall shear stress (WSS) levels decrease on the AAA wall [14,15]. WSS levels on the arterial wall are critical because the shear forces on the wall are sensed by the endothelial cells that are responsible for the remodeling process of artery [1,16]. Therefore, the deteriorated levels of WSS result in abnormal arterial remodeling and deposition of intraluminal thrombus (ILT) due to an increased relative residence time (RRT) of blood cells near the arterial wall [17,18].

In addition to WSS, other hemodynamic markers such as time-averaged WSS (TAWSS), oscillatory shear index (OSI), and endothelial cell activation potential (ECAP) are computationally investigated by the researchers [19,20]. TAWSS is the temporal average of WSS levels through one complete cardiac cycle. OSI is a measure to determine the directionality of the shear forces, as the low OSI indicates a unidirectional flow and high OSI points out a high variability in shear force directions [4]. ECAP is a metric that describes the potential of ILT deposition. As a general outcome, it is reported that low levels of TAWSS, high levels of OSI, and high levels of ECAP are the factors that increase the risk of AAA rupture in the long term [1].

The mechanism of AAA rupture is quite complex due to different material layers and uncertainties in the material properties on the AAA wall [21]. The wall structure consists of intima, media, and adventitia layers of the artery [22,23,24]. In addition to these arterial layers, calcified deposits and ILT volumes on the wall also affect the total strength of the AAA wall [25,26]. Medical imaging tools are commonly employed to obtain the exact spatial resolution of problem geometry, but the material properties of the structural layers cannot be determined by these imaging modalities. Therefore, the assessment of rupture [27,28] includes a patient-specific uncertainty depending on the unknown material properties in the AAA.

In several studies, the inclusion of ILT provided improved solution accuracy in terms of the mechanical stresses on the wall [29,30]. Mechanical stress can be considered as the most important factor in determining the risk of rupture because the rupture occurs when the mechanical stresses that arise due to the flow-induced hemodynamic forces are lower than the ultimate stress of AAA wall [27,31]. Therefore, determining the peak mechanical stresses has utmost importance to predict an impending mechanical failure on AAA wall.

In this study, a comprehensive computational analysis is performed to reveal the effects of ILT deposition and material properties on the AAA wall. For this purpose, FSI analyses are conducted by combining the hemodynamic results of CFD simulations with the structural AAA models. The generated models are based on a patient-specific AAA geometry that is determined by medical images. Various amounts of ILT burdens with different volumes are artificially generated inside the AAA models, and different material properties are employed for the artery and ILT structures. The results provided important insight to better understand the effect of ILT burden and material properties on the rupture risk of AAA.

## 2. Materials and Methods

In this study, the mechanical stresses on the artery are investigated considering three different parameters, namely, the presence of various ILT volumes in AAA, different material properties of ILT, and different material properties of the artery. Four different ILT burdens, three different ILT materials, and five different artery materials are employed in the analysis. In total, 50 different AAA models are artificially generated using a patient-specific geometry. The generated models are summarized in Table 1. The employed ILT and artery materials are explained in Section 2.3.1. In order to determine the peak mechanical stresses on the artery, FSI analyses are performed by coupling the structural models of AAA with the results of CFD simulations. CFD simulations and FSI coupling processes are performed using ADINA 9.7 software package (ADINA R&D Inc., Framingham, MA, USA).

### 2.1. Model Geometry

**Figure 1:**
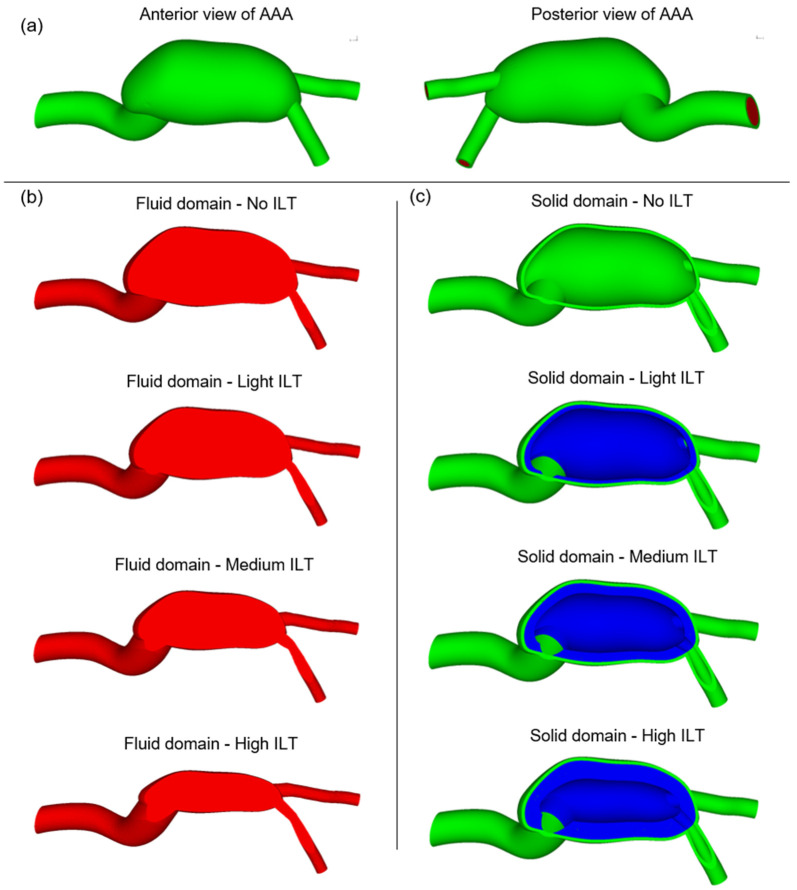
(**a**) Anterior and posterior views of the patient-specific AAA model. (**b**) The cross-sectional views of the fluid domains for four different ILT burdens, namely, No ILT, light ILT, medium ILT, and high ILT burdens. The fluid domain consists of blood. (**c**) The cross-sectional views of the solid domains for four different ILT burdens, namely, No ILT, light ILT, medium ILT, and high ILT burdens. The solid domain consists of the ILT and artery, which are shown in blue and green, respectively.

The solid domain of AAA models consists of the artery and ILT, while the fluid domain consists of blood. The fluid domain of the AAA model is extracted using the open source medical data of a 60-year-old male [32]. The fluid domain is surrounded by a 1.5 mm thick arterial structure [33,34]. In the original data, there is no ILT in the AAA; however, in order to elucidate the effect of ILT burden, three different volumes of ILT are artificially generated in the AAA. Thus, four different ILT formations are considered to model the ILT burden, namely, No ILT, light ILT, medium ILT and high ILT. The No ILT case does not have an ILT volume inside the AAA. The patient-specific AAA geometry and four different ILT formations are presented in Figure 1. The generated AAA geometries include the bifurcation of two iliac arteries at the bottom of the models.

The inlet and outlet boundaries of the AAA geometry are not ideal circles and resemble ellipses. Therefore, the distances between the edge points on the inlet and outlet boundaries exhibit certain variations in terms of diameters. At the inlet boundary, the outer diameter of artery varies within the range of 16–19 mm. This arterial diameter range corresponds to an inlet flow diameter of 13–16 mm due to the arterial thickness of 1.5 mm. At the outlet boundary, the outer diameter of artery varies within 8.5–10 mm, corresponding to an outlet flow diameter of 5.5–7 mm.

At the mostly expanded section of AAA, the outer diameter of artery varies within the range of 45–48 mm. For the No ILT case, the maximum flow diameter varies within 42–45 mm. This maximum flow diameter reduces to 30–32 mm for the light ILT case, 24–26 mm for the medium ILT case, and 19–21 mm for the high ILT case.

### 2.2. Modeling of the Fluid Domain

The governing equations in the fluid domain are known as the Navier–Stokes and continuity equations which are provided in Equations (1) and (2), respectively [35]. In the given equations, v is the tensor of flow velocity, $\rho_{f}$ is the mass density of blood, t is time, $\tau_{f}$ is the tensor of fluid stress, w is the velocity of fluid domain coordinate system due to the deformed solid domain. The continuity equation (Equation (2)) guarantees that the mass flow rate at the inlet is equal to the mass flow rate at the outlet.

(1) $$\rho_{f}\frac{\partial v}{\partial t}+\rho_{f}\left(v-w\right)\cdot \nabla v-\nabla \cdot \tau_{f}=0$$

(2) ∇·v=0

The flow simulations are performed using ADINA-CFD module of ADINA 9.7 (ADINA R&D Inc., Framingham, MA, USA). The laminar flow solver is employed using a time increment of 0.022 s. Several CFD studies in the literature report that the laminar flow assumption can adequately capture the AAA flow hemodynamics [24,36,37]. Three sequential cardiac cycles are simulated in all modeled cases. The difference in the maximum outlet velocities between the first and second cardiac cycles of Case 1 is found to be less than 2%. When the second and third cardiac cycles of Case 1 are compared, it is found that the maximum outlet velocities are almost identical in the second and third cardiac cycles, indicating a difference that is close to zero. This demonstrates that the transient effects diminish within the first two cardiac cycles. As a result, the findings of the third cardiac cycle are used for further analysis. Each cardiac cycle has a time length of 1.1 s and divided into 50 equal time steps.

#### 2.2.1. Boundary Conditions in the Fluid Domain

The boundary conditions are adapted from the clinical studies in the literature [35]. A time-dependent inlet flow velocity profile is applied at the top of the fluid domain. The applied inlet flow velocity has a plug profile with the same magnitude across the entire inlet area. Instead of using a parabolic or Womersley flow profile, a plug flow profile is preferred at the inlet due to the considerable distance between the inlet and aneurysm sac. There is a distance of approximately 50 mm between the inlet and aneurysm entrance, which is approximately 3.5 times greater than the inlet flow diameter. It is considered that the relatively long distance between the inlet and aneurysm can result in an adequately developed flow at the entrance of the aneurysm.

**Figure 2:**
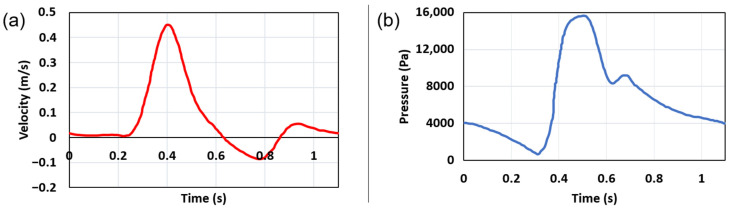
(**a**) Inlet velocity profile for one full cardiac cycle [35]. (**b**) Outlet pressure profile for one full cardiac cycle [35]. In the FSI simulations, the given profiles are applied during three sequential cardiac cycles.

At the two outlet surfaces of iliac arteries, a time-dependent pressure profile is applied. The inlet and outlet boundary conditions are provided in Figure 2. The outer surface of the fluid domain which contacts the solid domain is set with a no-slip boundary condition to guarantee that the flow velocity is zero on the AAA wall [1,4,9].

#### 2.2.2. Material Properties of Blood

Blood is modeled as a homogeneous, incompressible, and non-Newtonian fluid [38,39]. For non-Newtonian fluids, the local viscosity (μ) changes as a function of local shear rate ($\dot{\gamma }$). Therefore, the flow regions with different shear rates may have different local viscosities. According to the findings in the literature, non-Newtonian blood models provide better hemodynamic results in the cardiovascular models [40,41,42]. The Carreau viscosity model is employed for modeling the non-Newtonian behavior of blood, as given in Equation (3). The constants in Equation (3) are set as A=10.976, n=−0.3216, $\mu_{\infty }=0.0033$, and $\mu_{0}=0.056$ [43]. The mass density of blood is used as 1050 kg/m^3^ [44].

(3) $$\mu =\mu_{\infty }+\left(\mu_{0}-\mu_{\infty }\right){\left(1+A{\left|\dot{\gamma }\right|}^{2}\right)}^{n}$$

### 2.3. FSI Modeling

The hemodynamic results of fluid domain are coupled with solid models using ADINA-FSI module of ADINA 9.7 (ADINA R&D Inc., Framingham, MA, USA). For the data transfer between the fluid and solid domains, a two-way iterative coupling method is employed. In two-way coupling, the flow-induced forces are transferred to the solid domain [45]. The materials in the solid domain deform under the effect of transferred fluid-induced forces, and therefore, the geometric form of the solid changes. The new deformed state of the solid leads to a geometric change in the flow domain, meaning that the deformed geometric state in the solid domain is transferred to the fluid domain. This way, the solid and fluid domains counter-interactively transfer force and displacement at each time step of the FSI analysis.

#### 2.3.1. Governing Equations and Material Properties in Solid Domain

The momentum conservation given in Equation (4) is the governing equation in the solid domain. In Equation (4), $\tau_{s}$ implies the stress tensor of solid, $\rho_{s}$ indicates the mass density of solid, ${\ddot{d}}_{s}$ denotes the local acceleration of solid, and $f_{s}$ denotes the body forces on solid [35]. Due to the indiscernible effect of gravity, the gravitational forces ($f_{s}$) are neglected in the models.

(4) $$\nabla \cdot \tau_{s}+f_{s}=\rho_{s}{\ddot{d}}_{s}$$

Three different material models are used for the ILT and five different material models are employed for the artery. The reason for using various material models is the uncertainty in the material properties of the ILT and artery. The elastic modulus of the ILT is relatively lower than the arterial elastic modulus. When the ILT is initially formed, the elastic modulus is about 50 kPa [46]. The ILT structure becomes more rigid over time, eventually reaching a maximum stiffness of 200 kPa. Since the stiffness of ILT cannot be directly determined using the medical imaging tools, the elastic modulus of ILT is modeled using three different values—50 kPa, 100 kPa and 200 kPa. An elastic modulus of 50 kPa indicates a newly formed ILT. The value of 100 kPa indicates the nominal elastic modulus for the ILT, and 200 kPa indicates a relatively older ILT structure. As given in Table 2, ILT elastic modulus values of 50 kPa, 100 kPa, and 200 kPa are named as ILT material 1, ILT material 2, and ILT material 3, respectively. The Poisson’s ratio of ILT is used as 0.45 [47] and the mass density of ILT is used as 1050 kg/m^3^ [48] for all ILT material models.

A more detailed material model is employed for the artery using the hyperelastic Mooney–Rivlin model, since the rupture of artery is the main interest. The Mooney–Rivlin model has a nonlinear relationship between the mechanical stress and strain. The Mooney–Rivlin material model is defined in Equation (5), where W denotes the strain energy density function, $c_{1}$ and $c_{2}$ are empirically determined material constants, and $I_{1}$ and $I_{2}$ are the first and second invariants of the Cauchy–Green deformation tensor.

The studies in the literature showed that the values of $c_{1}$ and $c_{2}$ have certain variations from patient to patient [49]. The minimum, average, and maximum values of $c_{1}$ are reported as 15.2, 17.4, and 21.9 N/cm^2^, respectively. In a similar way, the minimum, average, and maximum values of $c_{2}$ are reported as 117.6, 188.1, and 355.7 N/cm^2^ [49]. The average $c_{1}$ and average $c_{2}$ values are considered as the nominal material model properties for the artery. By combining the given minimum, average, and maximum values of $c_{1}$ and $c_{2}$, five different material models are generated for the artery as summarized in Table 3. The Poisson’s ratio of artery is used as 0.49 [50] and the mass density is used as 2000 kg/m^3^ [51] for all artery material models.

(5) $$W=c_{1}\left(I_{1}-3\right)+c_{2}\left(I_{2}-3\right)$$

#### 2.3.2. Boundary Conditions in Solid Domain

The two ends of the artery, which correspond to the inlet and outlet sections, are fixed with zero displacement and zero velocity. The inner surface of artery and the outer surface of ILT contact to the fluid domain; therefore, these surfaces are selected as FSI boundaries in the solid domain [1]. Similarly, the outer surface of fluid domain contacts the artery and ILT; therefore, these surfaces in fluid domain are set as FSI boundaries in the fluid domain. The FSI surfaces of fluid and solid domains must perfectly match each other; otherwise, compatibility errors are observed during the force and displacement transfer between the fluid and solid domains. The surfaces between the ILT and artery are modeled as bonded surface boundaries, which assume that the ILT structure is bonded to the arterial structure.

## 3. Results

The maximum displacement magnitudes and maximum effective stresses on the artery are determined for 50 different AAA models presented in Table 1. In order to show the accuracy of the results, mesh independency studies are performed in the fluid and solid domains.

### 3.1. Mesh Independency in Fluid Domain

Three different fluid mesh densities are used to solve a sample case to show the mesh independence of the results. The sample case is selected as the Case 1 presented in Table 1. The reason for selecting Case 1 as the sample case is the nominal arterial material properties with no ILT deposition in the AAA sac which result in high variations in the expected hemodynamic findings. Since the artery wall has only experienced minor deformations of around 1 mm, the solid domain is assumed to be rigid for the mesh independence studies in the fluid domain.

The coarse, moderate, and dense fluid meshes have total element numbers of 58,641, 167,879, and 482,929, respectively. In all meshes, four-noded tetrahedral elements are employed. The peak pressure values in the third cardiac cycle are compared between these three meshes. According to the results, the difference of maximum pressure between the coarse and moderate fluid meshes is found as 0.42%, and the difference between the moderate and dense fluid meshes is determined as 0.1%. Since the difference in pressures between the moderate and dense meshes is lower than 1%, the results of the moderate fluid mesh are considered as satisfactorily accurate [52].

### 3.2. Mesh Independency in Solid Domain

Three different solid meshes are employed to test the mesh independence in the solid domain. For the mesh independency studies in the solid domain, the Case 41 presented in Table 1 is selected as the sample case due to the expected high solid deformations. The peak mechanical stresses are determined for Case 41, which has high ILT deposition with nominal ILT and nominal artery material properties. For the fluid mesh, previously determined moderate fluid mesh is employed.

The results are determined for the coarse, moderate and dense solid meshes, which correspond to total element numbers of 25,134 (16,337 for ILT and 8797 for artery), 138,148 (75,758 for ILT and 62,390 for artery), and 238,927 (154,264 for ILT and 84,663 for artery), respectively. The unit element sizes are set as 2 mm, 1 mm, and 0.75 mm for the coarse, moderate, and dense solid meshes, respectively. The solid elements are used as eight-noded hexahedral elements. The difference in peak mechanical stress between the coarse and moderate solid meshes is determined as 7.98%, and the difference in moderate and dense solid meshes is found as 0.86%. Since the difference between the moderate and dense solid meshes is less than 1%, the results of the moderate solid mesh are considered to be satisfactorily accurate [52]. Therefore, the moderate fluid mesh and the moderate solid mesh are used together for further FSI analyses.

### 3.3. Determination of Effective Stresses

The mechanical stresses on the arterial structure play an important role on the vascular remodeling [53] and they are the main indicators of an impending AAA rupture. The fundamental types of mechanical stresses can be listed as compression, tension, shear, torsion, and bending stresses [54]. To predict the failure of a structure, a number of mechanical failure criteria have been developed based on the mechanical stresses [54]. According to the Huber–von Mises–Hencky theory, the mechanical failure is expected if the square of von Mises stress ($\sigma_{s}$) is greater than the square of the uniaxial failure strength of the material. Therefore, the effective stresses are calculated using the formulation of von Mises stress ($\sigma_{s}$) provided in Equation (6), where $\sigma_{1}$, $\sigma_{2}$, $\sigma_{3}$ are the local principal stresses of the Cauchy stress tensor [35].

(6) $$\sigma_{s}=\sqrt{\frac{1}{2}[{\left(\sigma_{1}-\sigma_{2}\right)}^{2}+{\left(\sigma_{2}-\sigma_{3}\right)}^{2}+{\left(\sigma_{3}-\sigma_{1}\right)}^{2}]}$$

### 3.4. The Maximum Effective Stresses on Artery

The maximum effective stresses on artery are obtained at the instant of peak inlet flow rate. The maximum effective stresses on artery are presented for 50 different AAA models in Table 4. The determined stress levels show variations depending on the ILT burden, ILT material, and artery material. The levels of maximum effective stresses are approximately within the range of 510–730 kPa.

**Figure 3:**
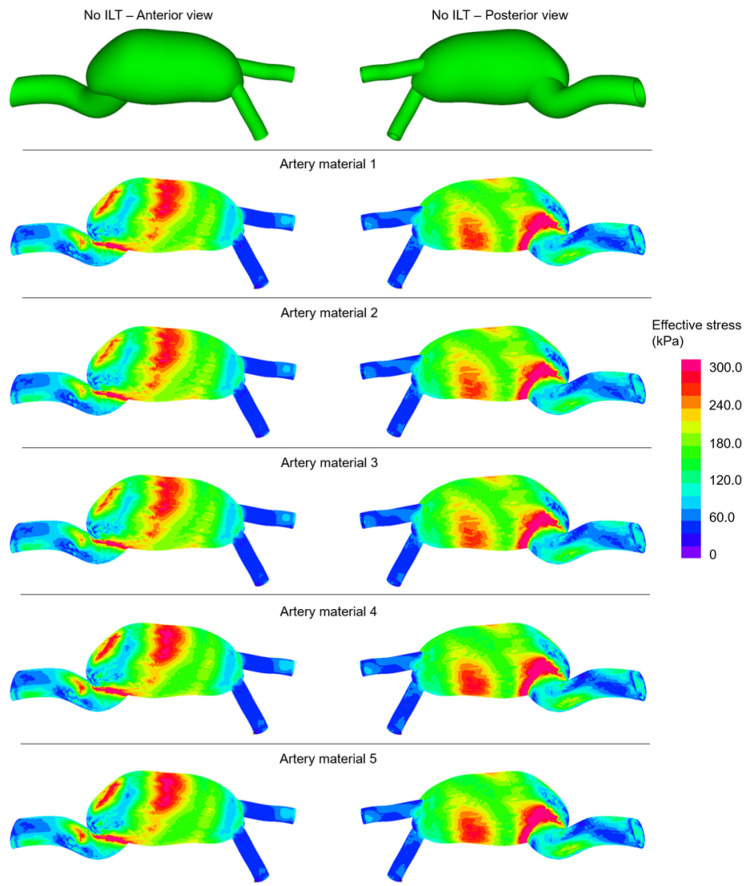
Effective stress distributions for the No ILT cases at the instant of peak inlet flow rate. The contour plots are provided from the anterior and posterior views considering five different artery materials.

In Figure 3, the distributions of effective stresses are shown for the No ILT cases at the instant of peak inlet flow rate. The colored contour plots are provided from the anterior and posterior views. For five different artery materials, similar stress distributions are observed in terms of the amplitudes. Three main high-stress locations are observed in all modeled cases, which are the anterior mid-section, the posterior mid-section, and the junction between the main aorta and AAA sac.

**Figure 4:**
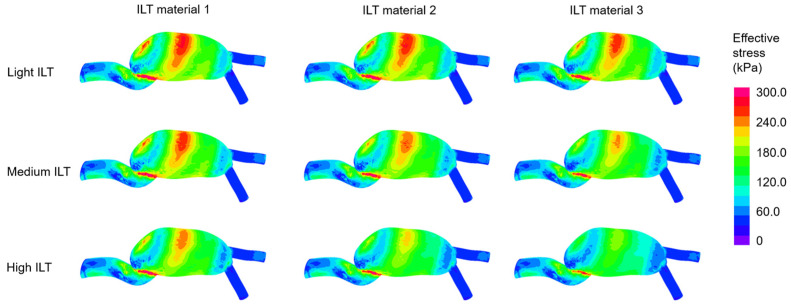
Effective stress distributions at the instant of peak inlet flow rate for various ILT burdens and ILT materials. The contour plots are provided from the anterior view. Artery material 2 is employed for the given 9 contour plots.

In Figure 4, the effective stress contour plots are provided considering different ILT material properties and ILT burdens. Artery model 2 is employed for all models presented in Figure 4. It is observed that the critical locations with high effective stress are coincident with the critical locations in the No ILT models. The increased ILT burden decreases the maximum stress on AAA. In a similar way, the increased ILT stiffness leads to decreased stress levels on the artery wall.

### 3.5. The Maximum Displacement Magnitudes on Artery

The maximum displacement magnitudes on artery are obtained at the instant of peak inlet flow rate. In Table 5, the maximum displacement magnitudes on artery are presented for 50 different AAA models. The determined maximum displacements show variations depending on the ILT burden and material models in the solid domain. The levels of maximum displacement are approximately within the range of 0.6–2.1 mm.

**Figure 5:**
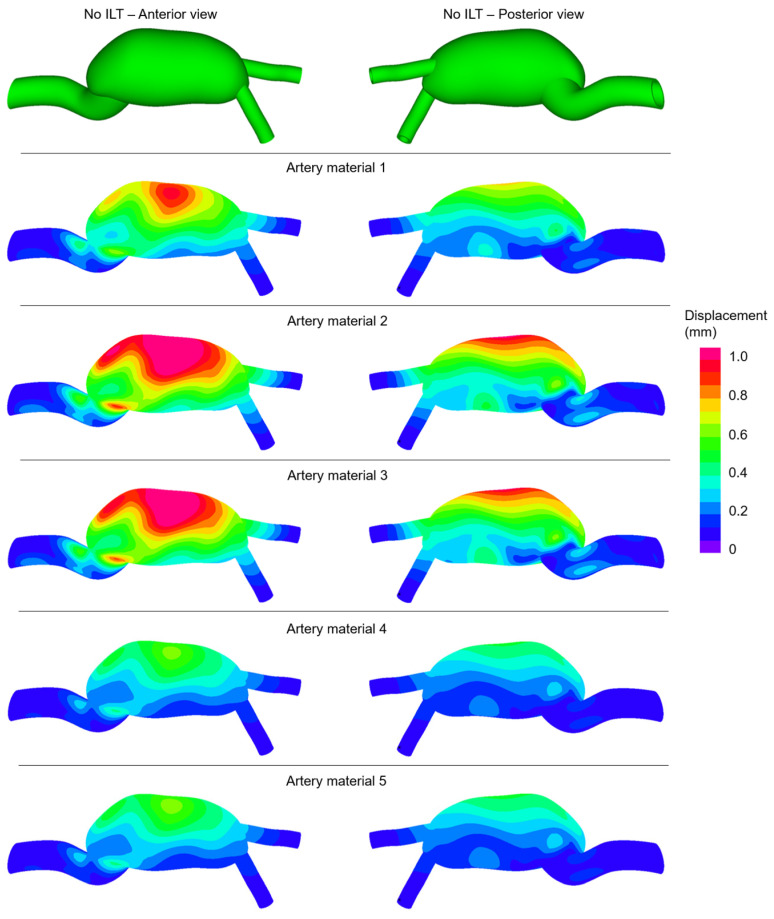
Displacement magnitude distributions for the No ILT cases at the instant of peak inlet flow rate. The contour plots are provided from the anterior and posterior views considering five different artery materials.

In Figure 5, the distributions of arterial displacements are given for the No ILT cases at the instant of peak inlet flow rate. In contrast to the stress distributions in the No ILT cases, the maximum displacement magnitudes show a high variance depending on the artery materials.

**Figure 6:**
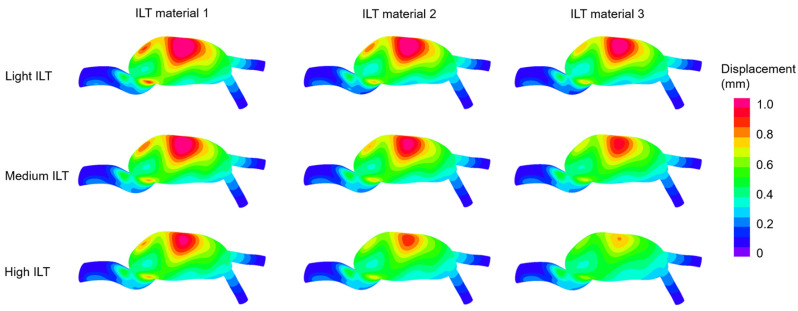
Displacement magnitude distributions at the instant of peak inlet flow rate for various ILT burdens and ILT materials. The contour plots are provided from the anterior view. Artery material 2 is employed for the given 9 contour plots.

In Figure 6, the displacement contour plots are provided considering different ILT material properties and ILT burdens. Artery model 2 is employed for all models presented in Figure 6. In all cases, the highest displacements are observed around the anterior mid-section. Similar to the findings of the effective stress, the increased ILT burden and increased ILT stiffness result in decreased arterial displacements.

### 3.6. Statistical Analyses on Maximum Effective Stresses

Statistical analyses are performed for the maximum effective stresses on artery by employing two tailed Student’s *t* test with paired data. Statistical significance is considered for the *p* values which are less than 0.05. The *p* values less than 0.005 are considered to be highly statistically significant.

The effect of artery material properties is investigated by employing five different artery material models. For No ILT case, the averages of maximum effective stresses are obtained as 719.8, 715.4, 714.5, 730.7, and 730.5 kPa for the five different artery materials. For light ILT cases, the averages of maximum effective stresses are determined as 663.5, 659.2, 658.7, 663.3, and 663.5 kPa for the artery materials of 1, 2, 3, 4, and 5, respectively. For medium ILT cases, these averages are found as 611.5, 610.7, 609.6, 638.4, and 636.5 kPa. In similar way, for high ILT cases, the averages of maximum effective stresses are calculated as 579.8, 563.8, 564.9, 595.2, and 595.0 kPa for the artery materials of 1, 2, 3, 4, and 5, respectively. The percent differences in the averages of peak stresses reach up to 1.5% for No ILT, 0.7% for light ILT, 4.4% for medium ILT, and 2.7% for high ILT cases. These results indicate that the stress variations due to the artery material properties are in the range of 0.7–4.4% and a direct relationship is not observed depending on the ILT burden.

Using the given averages of maximum effective stresses for different artery materials, *p* values are found as 0.000024 between No ILT and light ILT cases, 0.0024 between light ILT and medium ILT cases, and 0.000093 between medium ILT and high ILT cases. These findings point out that the formation and growth of ILT have a highly significant effect on the arterial stresses, and therefore on the risk of AAA rupture.

The averages of maximum effective stresses which are determined for different artery materials are compared considering five different artery models. Artery material 1 has the nominal arterial properties; therefore, the statistical analyses are performed by comparing the results of other artery materials with the results of artery material 1. The comparison between artery material 1 and artery material 2 resulted in a *p* value of 0.151. The comparisons between artery materials 1 and 3, artery materials 1 and 4, and artery materials 1 and 5 resulted in *p* values of 0.097, 0.099, and 0.091, respectively. This indicates that the effect of artery material properties on effective stresses are not statistically significant. When a similar comparison is carried out for investigating the effect of ILT material properties on effective stresses, it is observed that the comparison between ILT material 1 and ILT material 2 resulted in a *p* value of 0.120 and the comparison between ILT material 2 and ILT material 3 resulted in a *p* value of 0.103. These findings show that the effect of ILT material properties on effective stresses is not statistically significant.

## 4. Discussion

The mechanical determinant of AAA rupture is the effective stress on the artery wall. Therefore, the investigation of the effective stresses has utmost importance for detecting an impending AAA rupture. The effective stress on AAA wall can change depending on the ILT burden, ILT material properties, and arterial strength of AAA. As a consequence, the effects of material properties and ILT burden are elucidated by analyzing various scenarios including different ILT burdens and material properties.

The range of maximum effective stresses on artery is determined as 714.5–730.7 kPa for No ILT, 649.5–671.2 kPa for light ILT, 580.2–676.6 kPa for medium ILT, and 513.4–624.3 kPa for high ILT cases. This finding indicates that increasing amount of ILT burden results in a broader range for the maximum effective stresses. The percent difference between the minimum and maximum values of the given ranges are found as 2.3% for No ILT, 3.3% for light ILT, 16.6% for medium ILT, and 21.6% for high ILT cases. Therefore, the potential differences in mechanical stresses tend to become more prominent with increasing ILT burden in the AAA sac.

The average of the maximum effective stresses for all different scenarios are found as 722.2 ± 3.6 kPa for No ILT, 661.6 ± 1.8 kPa for light ILT, 621.3 ± 8.0 kPa for the medium ILT, and 579.7 ± 9.5 kPa for high ILT cases. The increasing ILT volume shows a softening effect on the arterial wall. The softening effect in maximum effective stresses is determined as 8.4% for light ILT, 14.0% for medium ILT, and 19.7% for high ILT cases. These percent values are determined by comparing the maximum effective stresses of the No ILT case with the results of the ILT-formed cases, namely, the light ILT, the medium ILT, and the high ILT cases.

The effect of ILT material properties is investigated by comparing the average values of the maximum effective stresses for various cases. For light ILT models, the average of maximum stress is obtained as 668.8 kPa for ILT elastic modulus of 50 kPa, 662.2 kPa for ILT elastic modulus of 100 kPa, and 653.9 kPa for ILT elastic modulus of 200 kPa. For medium ILT cases, the averages of maximum stresses are determined as 653.7, 621.1, and 589.1 kPa for ILT elastic modulus values of 50, 100, and 200 kPa, respectively. In a similar way, the averages of maximum stresses are determined as 618.9, 579.3 kPa, 541 kPa for ILT elastic modulus values of 50, 100, and 200 kPa, respectively. It is apparent that the peak stresses on AAA wall reduce with increasing elastic modulus of the ILT structure. However, the effective stress reduction due to ILT material properties is limited when compared to the stress reduction due to ILT burden. For light ILT cases, the stress reduction due to ILT material properties is in the range of 1.0–1.3%. This stress reduction due to ILT material properties reaches up to 5.2% for medium ILT cases and 6.8% for high ILT cases, indicating an increasing stress reduction with the increasing ILT amount in AAA sac.

To provide a better understanding of the effects of material properties and ILT burden, statistical analyses are performed using the averages of the maximum effective stresses for various cases. According to the findings, the only statistically significant parameter for the effective stresses is found to be the ILT burden. It is seen that the artery material properties and ILT material properties do not have a statistically significant effect on the effective stresses on the arterial wall. This suggests that the inclusion of ILT volume in the AAA model is critical for improving the accuracy of the rupture risk assessment of the aneurysm.

Similar conclusions can also be drawn for arterial wall displacements. The average of the maximum displacements on artery is determined as 1.03 ± 0.17 mm for the No ILT, 0.92 ± 0.08 mm for the light ILT, 1.12 ± 0.09 mm for the medium ILT, and 1.28 ± 0.12 mm for the high ILT cases. For the light ILT cases, the average values of the maximum displacements are determined as 0.97, 0.91, and 0.88 mm for the ILT elastic modulus values of 50, 100, and 200 kPa, respectively. For the medium ILT, these averages of the maximum displacements are obtained as 1.37, 1.08, and 0.91 mm. For the high ILT models, the averages of maximum displacements are found as 1.76, 1.19, and 0.88 mm for ILT elastic modulus values of 50, 100, and 200 kPa, respectively. It is seen that the average values of maximum displacement are within the range of 0.88–1.76 mm and the effect of ILT material properties on arterial displacement is less than 1 mm. Therefore, the arterial displacements are considered to be less critical compared to the arterial stresses, and the maximum effective stresses on the artery are mainly investigated to assess the rupture risk.

To summarize, the formation of ILT significantly changed the effective stress levels on the arterial structure of AAA. The increased amount of ILT reduced the flow volume inside the AAA sac and decreased the effective stress levels on the arterial wall with a softening effect. The stated effect of ILT can be considered as a factor that reduces the rupture risk of the aneurysm. For the No ILT case, the relatively larger flow volume in the AAA sac resulted in increased arterial stresses. This implies that the larger flow volumes in the larger aneurysms are expected to result in increased wall stresses, and therefore, increased risk of rupture. The material properties of the artery are partially effective on the arterial stresses; however, this effect is not statistically significant. Similarly, the increasing stiffness of the ILT structure results in a slight reduction in arterial stresses, but this reduction is also not statistically significant. Although the artery material properties and ILT material properties have limited effects on the arterial stresses, these effects are not critical compared to the effect of ILT volume. Therefore, the presence and current state of the ILT structure should be considered in detail for an improved rupture risk assessment of the AAA.

## 5. Conclusions

In this study, realistic AAA models are computationally investigated by performing flow simulations combined with FSI analyses. The effective stresses on the arterial structure are elucidated to reveal the effects of the ILT burden, the ILT material properties, and the artery material properties. For this purpose, 50 different scenarios are modeled considering various ILT burdens and material properties. The results obtained provided important information for understanding the mechanical stresses on the arterial structure. The increasing ILT volume resulted in a softening effect as the high ILT deposition in AAA led to reduced effective stresses on the artery. In a similar way, increasing ILT stiffness resulted in slightly reduced arterial stresses. According to the statistical analyses, the ILT burden has a significant effect on the arterial stresses. The effects of artery material properties and ILT material properties on arterial stresses are not statistically significant and are limited compared to the effect of ILT burden. It is concluded that the presence and volume of the ILT have important effects in AAA models, and the inclusion of the exact ILT volume is critical to improve the accuracy of the rupture risk assessment of the aneurysm.

## Figures and Tables

**Table 1:** The modeled AAA cases in FSI simulations.

Case	ILT Burden	ILT Material	Artery Material	Case	ILT Burden	ILT Material	Artery Material
1	No ILT	1	1	26	Medium	2	1
2	No ILT	1	2	27	Medium	2	2
3	No ILT	1	3	28	Medium	2	3
4	No ILT	1	4	29	Medium	2	4
5	No ILT	1	5	30	Medium	2	5
6	Light	1	1	31	Medium	3	1
7	Light	1	2	32	Medium	3	2
8	Light	1	3	33	Medium	3	3
9	Light	1	4	34	Medium	3	4
10	Light	1	5	35	Medium	3	5
11	Light	2	1	36	High	1	1
12	Light	2	2	37	High	1	2
13	Light	2	3	38	High	1	3
14	Light	2	4	39	High	1	4
15	Light	2	5	40	High	1	5
16	Light	3	1	41	High	2	1
17	Light	3	2	42	High	2	2
18	Light	3	3	43	High	2	3
19	Light	3	4	44	High	2	4
20	Light	3	5	45	High	2	5
21	Medium	1	1	46	High	3	1
22	Medium	1	2	47	High	3	2
23	Medium	1	3	48	High	3	3
24	Medium	1	4	49	High	3	4
25	Medium	1	5	50	High	3	5

**Table 2:** Elastic modulus values of different ILT material models.

ILT Material Model	Elastic Modulus (kPa)
1	50
2	100
3	200

**Table 3:** The Mooney–Rivlin model constants of $c_{1}$ and $c_{2}$ for five different artery material models.

Artery Material Model	$\mathrm{Value}\;\mathrm{of}\;c_{1}$ (N/cm^2^)	$\mathrm{Value}\;\mathrm{of}\;c_{2}$ (N/cm^2^)
1	17.4	188.1
2	15.2	117.6
3	21.9	117.6
4	21.9	355.7
5	15.2	355.7

**Table 4:** The maximum effective stress on artery for 50 different AAA models.

Case	ILT Burden	ILT Material	Artery Material	Maximum Stress on Artery (kPa)	Case	ILT Burden	ILT Material	Artery Material	Maximum Stress on Artery (kPa)
1	No ILT	-	1	719.8	26	Medium	2	1	610.2
2	No ILT	-	2	715.4	27	Medium	2	2	610.2
3	No ILT	-	3	714.5	28	Medium	2	3	609.0
4	No ILT	-	4	730.7	29	Medium	2	4	639.1
5	No ILT	-	5	730.5	30	Medium	2	5	637.2
6	Light	1	1	670.1	31	Medium	3	1	588.3
7	Light	1	2	666.2	32	Medium	3	2	580.3
8	Light	1	3	665.6	33	Medium	3	3	580.2
9	Light	1	4	671.0	34	Medium	3	4	599.4
10	Light	1	5	671.2	35	Medium	3	5	597.3
11	Light	2	1	664.2	36	High	1	1	619.2
12	Light	2	2	661.7	37	High	1	2	613.6
13	Light	2	3	660.9	38	High	1	3	613.2
14	Light	2	4	661.9	39	High	1	4	624.2
15	Light	2	5	662.2	40	High	1	5	624.3
16	Light	3	1	656.1	41	High	2	1	579.5
17	Light	3	2	649.7	42	High	2	2	564.5
18	Light	3	3	649.5	43	High	2	3	565.5
19	Light	3	4	656.9	44	High	2	4	593.6
20	Light	3	5	657.1	45	High	2	5	593.4
21	Medium	1	1	635.9	46	High	3	1	540.6
22	Medium	1	2	641.6	47	High	3	2	513.4
23	Medium	1	3	639.5	48	High	3	3	516.0
24	Medium	1	4	676.6	49	High	3	4	567.7
25	Medium	1	5	674.9	50	High	3	5	567.2

**Table 5:** The maximum displacement magnitudes on artery for 50 different AAA models.

Case	ILT Burden	ILT Material	Artery Material	Maximum Displacement on Artery (mm)	Case	ILT Burden	ILT Material	Artery Material	Maximum Displacement on Artery (mm)
1	No ILT	-	1	1.03	26	Medium	2	1	1.09
2	No ILT	-	2	1.44	27	Medium	2	2	1.41
3	No ILT	-	3	1.38	28	Medium	2	3	1.37
4	No ILT	-	4	0.64	29	Medium	2	4	0.77
5	No ILT	-	5	0.65	30	Medium	2	5	0.78
6	Light	1	1	0.97	31	Medium	3	1	0.92
7	Light	1	2	1.33	32	Medium	3	2	1.21
8	Light	1	3	1.28	33	Medium	3	3	1.17
9	Light	1	4	0.62	34	Medium	3	4	0.63
10	Light	1	5	0.63	35	Medium	3	5	0.64
11	Light	2	1	0.91	36	High	1	1	1.76
12	Light	2	2	1.24	37	High	1	2	2.10
13	Light	2	3	1.20	38	High	1	3	2.05
14	Light	2	4	0.59	39	High	1	4	1.44
15	Light	2	5	0.59	40	High	1	5	1.45
16	Light	3	1	0.88	41	High	2	1	1.20
17	Light	3	2	1.19	42	High	2	2	1.48
18	Light	3	3	1.16	43	High	2	3	1.45
19	Light	3	4	0.57	44	High	2	4	0.92
20	Light	3	5	0.58	45	High	2	5	0.92
21	Medium	1	1	1.38	46	High	3	1	0.89
22	Medium	1	2	1.73	47	High	3	2	1.12
23	Medium	1	3	1.69	48	High	3	3	1.09
24	Medium	1	4	1.03	49	High	3	4	0.65
25	Medium	1	5	1.04	50	High	3	5	0.66

## Data Availability

Not applicable.
